# WHO Critical- and High-Priority Fungal Pathogens Beyond Human Medicine: Expanding One Health Surveillance

**DOI:** 10.3390/pathogens15070660

**Published:** 2026-06-23

**Authors:** Ricardo Lopes, Andreia Garcês, Vanessa Silva, Hugo Lima de Carvalho, Filipe Sampaio, Gonçalo Barros, Cátia Fernandes, Ana Patrícia Lopes, Cátia Marques, Luís Cardoso, Elsa Leclerc Duarte, Ana Cláudia Coelho

**Affiliations:** 1 CEDIVET Veterinary Laboratories, Lionesa Business Hub, R. Lionesa 446 C24, 4465-671 Leça do Balio, Portugal; vanessa.campos.silva@cedivet.pt (V.S.); hugo.carvalho@cedivet.pt (H.L.d.C.); filipe.sampaio@cedivet.pt (F.S.); goncalo.barros@cedivet.pt (G.B.); 2 Department of Veterinary Sciences, University of Trás-os-Montes e Alto Douro (UTAD), 5000-801 Vila Real, Portugal; aplopes@utad.pt (A.P.L.); lcardoso@utad.pt (L.C.); accoelho@utad.pt (A.C.C.); 3 Department of Veterinary and Animal Sciences, University Institute of Health Sciences (IUCS), CESPU, 4585-116 Gandra, Portugal; 4 Wildlife Rehabilitation Centre (CRAS), Veterinary Teaching Hospital, University of Trás-os-Montes e Alto Douro (UTAD), 5000-801 Vila Real, Portugal; andreiamvg@gmail.com; 5 Animal and Veterinary Research Centre (CECAV), Associate Laboratory for Animal and Veterinary Sciences (AL4AnimalS), University of Trás-os-Montes e Alto Douro (UTAD), 5000-801 Vila Real, Portugal; catia.marques@ulusofona.pt; 6 Cytology and Hematology Diagnostic Services, Laboratory of Histology and Embryology, Department of Microscopy, ICBAS-School of Medicine and Biomedical Sciences, University of Porto (U. Porto), Rua de Jorge Viterbo Ferreira, 228, 4050-313 Porto, Portugal; 7 AniCura Santa Marinha Veterinary Hospital, R. Dom Henrique de Cernache 183, 4400-625 Vila Nova de Gaia, Portugal; catia.fernandes@anicura.pt; 8 I-MVET, Research in Veterinary Medicine, Faculty of Veterinary Medicine, Lusófona University, Lisbon University Centre, 1749-024 Lisbon, Portugal; 9 ECVM Satellite Training Centre, Genevet-INNO, 2790-140 Carnaxide, Portugal; 10 Department of Veterinary Medicine, School of Science and Technology, University of Évora, Polo da Mitra, Apartado 94, 7002-554 Évora, Portugal; emld@uevora.pt; 11 Mediterranean Institute for Agriculture, Environment and Development (MED), Global Change and Sustainability Institute (CHANGE), University of Évora, Polo da Mitra, Apartado 94, 7002-554 Évora, Portugal

**Keywords:** antifungal resistance, *Candida albicans*, *Cryptococcus neoformans*, One Health, ornamental birds, Planetary Health, Portugal, veterinary mycology, WHO Fungal Priority Pathogens

## Abstract

In 2022, the World Health Organization (WHO) published the Fungal Priority Pathogens List (FPPL), yet its application has remained largely focused on human medicine, with limited consideration of animal hosts and veterinary diagnostics. This retrospective study aimed to characterise the occurrence of WHO critical- and high-priority yeasts in veterinary clinical submissions in Portugal within an explicit One Health framework. All yeast-positive submissions received by a Portuguese veterinary diagnostic laboratory between 2019 and 2026 were reviewed. Isolates were identified phenotypically, by an automated identification system and by MALDI-TOF mass spectrometry. Data on host species, sample type and year of submission were analysed using standard descriptive and inferential statistics. Among 2033 mycological submissions, 219 were yeast-positive. Out of these, 82 isolates (37.4%) corresponded to WHO critical- or high-priority taxa, most frequently *Candida albicans*, followed by *Nakaseomyces glabratus*, *Cryptococcus neoformans* var. *neoformans*, the *Candida parapsilosis* complex and *Candida tropicalis*. The remaining 137 isolates (62.6%) corresponded to non-WHO taxa, among which the most frequent were *Papiliotrema laurentii* and *Debaryomyces hansenii* (*n* = 21 each; 15.3%), followed by the *Stephanoascus ciferrii* complex (*n* = 15; 11.0%), *Meyerozyma guilliermondii* and *Candida sake* (*n* = 12 each; 8.8%), and *Wickerhamomyces anomalus* (*n* = 9; 6.6%). WHO-prioritised taxa were recovered predominantly from ornamental birds, as well as from dogs, cats and marine mammals. These findings demonstrate that FPPL-listed yeasts are regularly detected among yeast-positive veterinary diagnostic submissions and highlight ornamental birds as prominent hosts within this dataset.

## 1. Introduction

Fungal pathogens recognised as critical or of high priority by the World Health Organization (WHO)—including *Aspergillus fumigatus*, *Candidozyma auris* (formerly *Candida auris*), *Candida albicans*, and *Cryptococcus neoformans*—are increasingly detected not only in human clinical settings but also in animals and diverse environmental reservoirs such as soil, water, agricultural fields, and wildlife [1,2,3,4,5,6,7,8]. Invasive fungal infections are rising worldwide, particularly among immunocompromised and critically ill patients, and surveillance studies have confirmed the presence of these pathogens in companion animals, livestock, wild birds (notably pigeons), bats, and even marine mammals, as well as in agricultural soils and urban environments [2,5,6,7,9].

**Figure 1:**
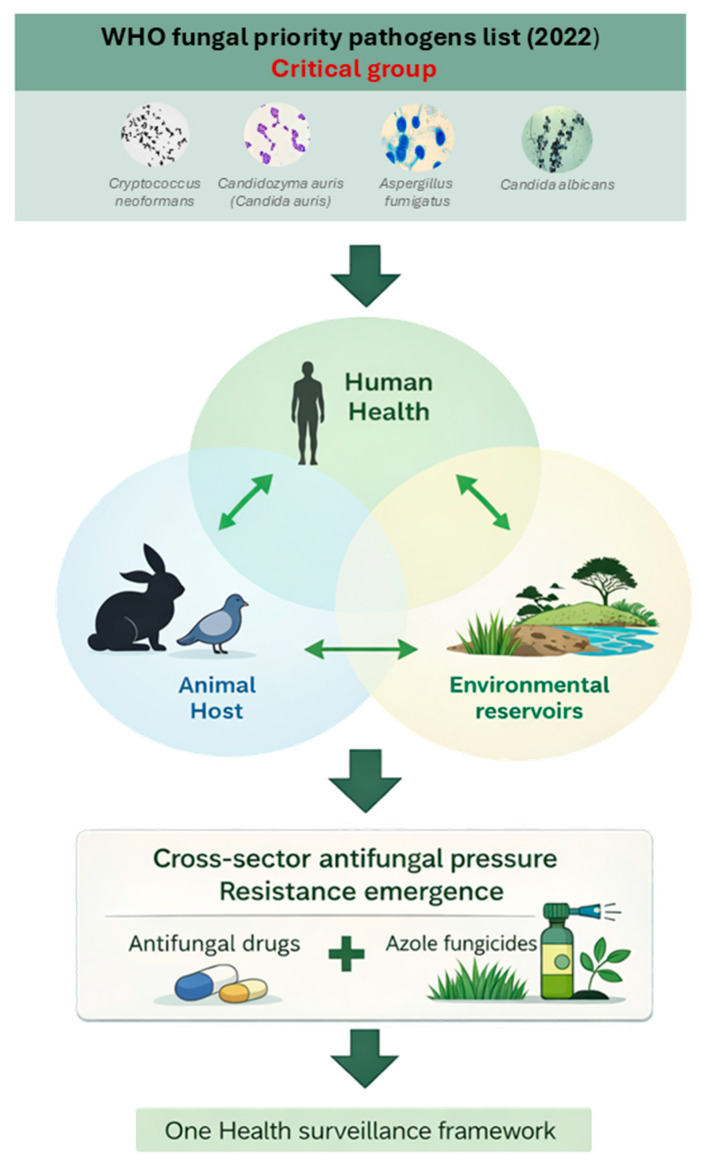
Conceptual One Health framework linking WHO priority fungal pathogens, animal hosts, environmental reservoirs, and cross-sector antifungal selection pressure. The diagram illustrates how WHO critical-priority fungal pathogens, including *Cryptococcus neoformans*, *Candidozyma auris* (*Candida auris*), *Aspergillus fumigatus* and *Candida albicans*, extend beyond human clinical settings and may involve animal hosts, environmental reservoirs and shared exposure interfaces. It also highlights how antifungal drug use and azole fungicides may generate cross-sector selection pressure, contributing to antifungal resistance emergence. Together, these interactions support the need for integrated One Health surveillance involving human medicine, veterinary medicine and environmental monitoring.

In response to these challenges, the WHO published the first WHO Fungal Priority Pathogens List (WHO FPPL) in 2022, representing the first global effort to systematically prioritise fungal pathogens according to unmet research and development needs and perceived public health importance [10,11]. This list ranked 19 fungal pathogens into critical-, high-, and medium-priority groups and was explicitly designed to guide surveillance, research investment, diagnostic development, antifungal innovation, and public health action [10,12,13]. Among the yeasts included in the critical- and high-priority categories are *Cryptococcus neoformans*, *Candida albicans*, *Nakaseomyces glabratus* (formerly *Candida glabrata*), *Candida tropicalis* and *Candida parapsilosis*, all of which are clinically important opportunists associated with substantial morbidity, mortality, and, in several cases, increasing antifungal resistance [10,13,14,15,16,17,18]. To contextualise these interconnections within a One Health perspective, a conceptual framework is presented in Figure 1.

In this context, animals should not be considered solely as incidental hosts. For several priority fungi, animal-derived detections may reflect clinically relevant infection, commensal or opportunistic colonisation, environmental exposure, or passive carriage. Each of these scenarios has different clinical implications, but all may be epidemiologically informative within a One Health framework, particularly when animals live in close contact with humans or share contaminated domestic, urban, agricultural or wildlife-associated environments.

Although the WHO FPPL constitutes a landmark advance in medical mycology, its conceptual architecture remains predominantly centred on human invasive disease. The prioritisation process focused chiefly on pathogens causing acute or subacute systemic infections in humans, with particular emphasis on mortality, annual incidence, complications and sequelae, antifungal resistance, access to diagnostics, and evidence-based treatment availability [10,13]. This focus is entirely justified from a public health perspective, yet it also means that the role of animals and environmental reservoirs in the ecology, persistence, dissemination, and possible evolution of these fungi remains insufficiently integrated into mainstream prioritisation and surveillance frameworks [8,12,13]. This limitation is especially relevant because fungal emergence, adaptation, and resistance are not phenomena confined to hospitals or human hosts, but unfold across interconnected human, animal, agricultural, and environmental systems, precisely the domains addressed by the Planetary Health paradigm [1,3,6,19,20]. Collectively, the current evidence suggests that non-human reservoirs may contribute to the persistence and circulation of medically important fungi, while animals may serve as sentinels of shared environmental exposure [21,22,23]. These patterns also reinforce concern over the cross-sectoral selection and dissemination of antifungal resistance, particularly in relation to antifungal and azole fungicide use across medical, veterinary, and agricultural settings [1,2,4,5,24,25].

Within this broader framework, priority yeasts deserve particular attention. *C. neoformans*, a WHO critical-priority pathogen, is a major environmentally acquired yeast associated with severe invasive disease, especially cryptococcal meningoencephalitis [8,10]. *C. albicans*, also classified as a critical priority, remains one of the most important opportunistic fungal pathogens worldwide, reflecting its dual role as both commensal and pathogen [10,16,19,20]. High-priority yeasts, including *N. glabratus*, *C. tropicalis*, and *C. parapsilosis*, are increasingly relevant because of their role in invasive candidiasis, their adaptation to vulnerable hosts and healthcare-associated environments, and their association with reduced antifungal susceptibility and multidrug resistance [10,14,15,16,26,27]. Importantly, these yeasts may behave as commensals, colonisers, opportunists, or true pathogens depending on the host, anatomical site, microbial burden, and immune status, which is particularly relevant when interpreting veterinary diagnostic samples [6,15,16]. From a veterinary perspective, this distinction is critical. The detection of a WHO-prioritised yeast in an animal-derived sample should not automatically be equated with clinically significant disease, nor should it be interpreted as direct evidence of zoonotic transmission. Nevertheless, such findings remain scientifically valuable, as they provide insight into host breadth, ecological distribution, anatomical niches, and shared environmental interfaces, and may help identify underrecognised reservoirs or sentinel populations [1,6,25,28,29]. Moreover, the relevance of animal mycology extends beyond individual case management. Fungal pathogens affect companion animals, livestock, and wildlife, contribute to biodiversity loss and ecosystem disruption, and intersect with human health through shared environments, exposure pathways, and resistance ecology [3,6,8,14,19,20,25,28,29]. The One Health significance of fungal disease, therefore, lies not only in direct spillover, but also in the interconnected processes of circulation, adaptation, environmental persistence, and therapeutic selection that link human, animal, and environmental health [1,3,6,19,20,24,25,30]. Despite substantial advances in medical mycology, systematic data on animal hosts and non-clinical reservoirs remain limited, fragmented, geographically uneven, and methodologically heterogeneous [1,6,25,28]. As a result, the animal dimension of the WHO FPPL remains insufficiently characterised, and the potential contribution of veterinary diagnostic systems to fungal surveillance has likely been underestimated [1,10,25,28].

The present study aims to address this knowledge gap by reviewing and analysing the occurrence of WHO critical- and high-priority yeasts in veterinary diagnostic submissions from Portugal. To the best of the authors’ knowledge, this is the first comprehensive Portuguese study to investigate these fungal pathogens within an explicit One Health surveillance framework. Rather than inferring direct zoonotic transmission or population-level prevalence, the objective is to document the occurrence of medically important yeasts across animal hosts and specimen types, thereby highlighting an underrecognised veterinary and environmental dimension in the ecology of WHO-prioritised fungal pathogens. Accordingly, this study should be interpreted as a retrospective description of occurrence among veterinary diagnostic submissions, rather than as an estimate of disease occurrence, active infection, subclinical carriage or risk of zoonotic transmission.

## 2. Materials and Methods

### 2.1. Data Collection, Sampling, and Microbiological Analysis

All clinical submissions for mycological culture received by CEDIVET Veterinary Laboratories (Porto, Portugal) between 2019 and 2026 were initially reviewed for eligibility. These submissions originated from veterinary practices, including clinics and hospitals, located across mainland Portugal and the autonomous insular regions. For the purposes of the present study, only records yielding yeast-like fungal isolates were included in the final analysis. No active population-based screening was performed. Each submission included a standard laboratory requisition form, which provided clinical data for each animal, including species, breed, sex, age, relevant medical history, vaccination and prophylactic status, clinical suspicion or observed signs (e.g., nasal discharge, neurological deficits, cutaneous lesions), and requested diagnostic tests.

Samples were directly inoculated onto Sabouraud Dextrose Selective Agar, Emmons formulation, supplemented with chloramphenicol (50.0 mg/L) and gentamicin (40.0 mg/L) (Thermo Scientific™, R01772, Waltham, MA, USA), and incubated at 30 °C. Culture plates were examined daily for fungal growth for up to 14 days, or until sufficient growth was obtained for identification. Phenotypic identification of fungal isolates was based on both macroscopic and microscopic characteristics. Macroscopic evaluation included assessment of colony growth rate, topography, texture, surface and reverse pigmentation, and the presence and colour of any diffusible pigment. Reverse pigmentation was defined as the colour observed on the underside of the fungal colony when viewed through the culture medium. Microscopic identification was performed using Gram staining (Previ Color Gram, bioMérieux, Marcy l’Étoile, France), evaluating yeast cell morphology, including cell shape, size, budding pattern, and the presence of pseudohyphae.

Pure yeast colonies were initially identified using the automated VITEK^®^ 2 XL system with the VITEK^®^ 2 YST identification card (ref. 21343; bioMérieux S.A., Marcy-l’Étoile, France), following the manufacturer’s instructions and validated interpretive criteria. For confirmatory species-level identification, pure colonies were subsequently analysed by matrix-assisted laser desorption/ionisation time-of-flight mass spectrometry (MALDI-TOF MS) using the microflex^®^ LT/SH system (Bruker Daltonics, Bremen, Germany). MALDI-TOF MS identifications were accepted according to the manufacturer’s score thresholds [31,32]. Taxa were classified according to the WHO Fungal Priority Pathogens List (WHO FPPL) [11]. For the purposes of this study, critical- and high-priority yeasts included *C. albicans*, *C. neoformans*, *N. glabratus*, *C. tropicalis* and the *C. parapsilosis* complex.

In the context of this retrospective study, the analytical unit was the individual microbiological record, with each record corresponding to a single yeast isolate recovered from one unique animal. No repeated submissions from the same animal were present in the final dataset.

Yeast isolates were interpreted as microbiological recoveries from veterinary diagnostic submissions and not necessarily as confirmed aetiological agents of disease. Because this was a retrospective laboratory-based study, clinical causality was not assigned solely based on culture positivity. Interpretation as commensal carriage, colonisation, opportunistic infection or clinically relevant fungal disease depends on host species, anatomical site, sample type, fungal burden and clinical context, which were not uniformly available for all records.

Records were included when a yeast-like fungal isolate was recovered and identified to species or species-complex level, irrespective of whether the isolate could be clinically classified as colonisation or clinically relevant infection. Records were excluded when no fungal growth was obtained, when only filamentous fungi were recovered, when identification was incomplete, or when duplicate isolates from the same animal were present.

### 2.2. Statistical Analysis

All available data were extracted in digital format from the Sislab^®^ system (Glintt, Global Intelligent Technologies, Lisbon, Portugal) and transferred to Microsoft Excel^®^ (Microsoft Corp., Redmond, WA, USA) for preprocessing. Categorical variables included animal host group, sample type, sex, year of submission, fungal pathogen classification according to the WHO FPPL, and microbiological outcome, whereas age was retained as a continuous variable and analysed in years.

Statistical analysis was performed using JMP^®^ Pro version 18.0.2 (SAS Institute Inc., Cary, NC, USA), numiqo (numiqo Team, 2026; numiqo e.U., Graz, Austria), and MedCalc^®^ statistical software version 20.006 (MedCalc Software Ltd., Ostend, Belgium). Descriptive statistics summarised counts and proportions. Associations between the WHO FPPL classification and categorical variables were assessed using the chi-squared (χ^2^) test, Fisher’s exact test or the Fisher–Freeman–Halton exact test when appropriate. Given the non-normal distribution of age, groups were compared using the two-tailed Mann–Whitney U test. A *p*-value ≤ 0.05 was considered statistically significant [33].

## 3. Results

### 3.1. Microbiological Study of WHO Priority Fungal Pathogens in Portugal Between 2019–2026

#### 3.1.1. General Recovery and Specimen Distribution

A total of 2033 fungal culture samples were submitted during this period. Of these, 219 yeast-positive microbiological records were included in this study. During the study period, a total of 82 isolates (37.4%; 95% CI: 31.0–44.2%) corresponded to WHO critical- or high-priority fungal pathogens, whereas 137 isolates (62.6%; 95% CI: 55.8–69.0%) corresponded to non-WHO-prioritised yeast taxa. The 82 WHO-priority recoveries comprised *C. albicans* (*n* = 38), *N. glabratus* (*n* = 20), *C. neoformans* var. *neoformans* (*n* = 12), *C. parapsilosis* complex (*n* = 10), and *C. tropicalis* (*n* = 2). Among the 137 non-WHO yeast isolates, the most frequently recovered taxa were *Papiliotrema laurentii* (formerly *C. laurentii*) and *Debaryomyces hansenii* (formerly *C. famata*) (*n* = 21 each; 15.3%), followed by the *Stephanoascus ciferrii* complex (formerly *C. ciferrii*) (*n* = 15; 11.0%), *Meyerozyma guilliermondii* (formerly *C. guilliermondii*) and *Candida sake* (*n* = 12 each; 8.8%), and *Wickerhamomyces anomalus* (formerly *Candida pelliculosa*) (*n* = 9; 6.6%). The complete distribution of non-WHO taxa is provided in Supplementary Table S1. The distribution of WHO critical- and high-priority fungal pathogens across host groups is detailed in Table 1.

WHO-priority pathogens were recovered predominantly from avian hosts (*n* = 57/152; 37.5%), followed by canines (*n* = 12/35; 34.3%), felines (*n* = 8/20; 40%), and marine mammals (*n* = 5/5; 100%). No WHO critical- or high-priority pathogens were recorded in equines (*n* = 0/2; 0.0%), lagomorphs (*n* = 0/1; 0.0%), or non-mammalian non-avian vertebrates (*n* = 0/4; 0.0%; reptiles and fish).

The relative distribution of sample types differed between WHO-priority and non-WHO yeast recoveries (Fisher–Freeman–Halton exact test, *p* = 0.004), indicating that WHO-priority yeasts were not uniformly distributed across specimen categories. However, this association should be interpreted cautiously because several sample types were represented by small counts. Overall, among the 82 WHO-priority recoveries, faecal swabs predominated (*n* = 43), followed by cutaneous swabs (*n* = 15), oropharyngeal swabs (*n* = 12), ear swabs (*n* = 4), organ tissue samples (*n* = 3), urine (*n* = 2), and single recoveries from nasal/ocular, air sac, and spiracle swabs (*n* = 1 each) (Table 2). When stratified by host category, the 57 avian WHO-priority recoveries comprised faecal swabs (*n* = 43), oropharyngeal swabs (*n* = 9), cutaneous swabs (*n* = 4), and one air sac swab (*n* = 1). In marine mammals, the five recoveries originated from oropharyngeal swabs (*n* = 3), one spiracle swab, and one nasal/ocular swab.

#### 3.1.2. Animal Species and Breeds

As shown in Table 1, the proportion of WHO critical- or high-priority yeasts within each host group category was 37.5% in avian species (57/152), 34.3% in canines (12/35), 40.0% in felines (8/20), and 100.0% in marine mammals (5/5), whereas no WHO-priority yeasts were recorded in equines (0/2), lagomorphs (0/1), or non-mammalian non-avian vertebrates (0/4). For inferential analysis, the Fisher–Freeman–Halton exact test across the seven host species categories indicated a statistically significant association between host species and WHO-priority recovery (*p* = 0.005). However, this result should be interpreted cautiously because the contingency table was sparse, several categories were represented by very small denominators, and the marine mammal category comprised only five records, all of which yielded WHO-priority yeasts. Accordingly, these findings should be regarded as exploratory and should not be interpreted as robust evidence of a stable host-category-specific risk.

Among canine and feline WHO-priority recoveries, cases were concentrated in a limited number of breeds, all represented by small denominators (Table 3). In dogs, mixed-breed animals accounted for most recoveries (*n* = 8), followed by Boston Terriers (*n* = 2) and German Shepherds (*n* = 2). In cats, most recoveries were recorded in European domestic cats (*n* = 7), with one additional case in a Persian cat. Given the low number of submissions within most breed categories, these findings should be regarded as descriptive and should not be interpreted as evidence of breed predisposition. All marine mammal WHO-priority recoveries were obtained from common dolphins (*Delphinus delphis*).

Across avian hosts, WHO-priority recoveries were concentrated in a relatively limited number of ornamental and captive taxa, particularly cockatiels (*Nymphicus hollandicus*) (*n* = 10), African grey parrots (*Psittacus erithacus*) (*n* = 7), lovebirds (*Agapornis roseicollis*) (*n* = 7), domestic canaries (*Serinus canaria*) (*n* = 4), white cockatoos (*Cacatua alba*) (*n* = 3), and Alexandrine parakeets (*Psittacula eupatria*) (*n* = 3), with the remaining recoveries distributed across multiple additional avian species (Table 4). Within avian WHO-priority recoveries, *C. albicans* was the predominant agent (33/57; 57.9%), followed by *N. glabratus* (18/57; 31.6%), *C. parapsilosis* complex (5/57; 8.8%), and *C. tropicalis* (1/57; 1.8%).

#### 3.1.3. Sex

Of the 219 fungal-positive microbiological records included in this study, sex information was available for 118 (53.9%). For the remaining 101 records (46.1%), sex was not specified on the submission form, and these were therefore excluded from the analysis. Among the 118 records with available sex data, 84 corresponded to males and 34 to females. No significant association was identified between sex and WHO critical- or high-priority fungal pathogen recovery (Fisher’s exact test, *p* = 0.835).

#### 3.1.4. Age

From the 219 fungal-positive microbiological records included in this study, age information was available for 44 records (20.1%), whereas the remaining 175 records (79.9%) lacked age data and were therefore excluded from the age-based analysis. Because the dataset comprised multiple host species categories, including avian, canine, feline, marine mammal, equine, lagomorph, and non-mammalian non-avian vertebrate records, age was analysed both in aggregate and, where possible, within individual host species categories. Of these 44 records, 15 yielded WHO critical- and high-priority fungal pathogens and 29 yielded non-WHO fungal pathogens. Records yielding WHO critical- and high-priority pathogens ranged from 0.5 to 14.0 years, with a median age of 9.0 years (IQR: 5.0–13.0 years), whereas records yielding non-WHO fungal pathogens ranged from 0.5 to 17.0 years, with a median age of 6.0 years (IQR: 1.0–9.0 years). Given the non-parametric distribution of age, a Mann–Whitney U test was applied, revealing no statistically significant difference in age between records yielding WHO critical- and high-priority pathogens and those yielding non-WHO fungal pathogens (U = 166.5, z = −1.27, exact *p* = 0.213, *r* = 0.19). Likewise, no statistically significant association with age was identified either in the overall dataset or within any individual host species category analysed.

#### 3.1.5. Year of Submission

Among the 82 WHO critical- and high-priority fungal pathogen recoveries, the annual distribution peaked in 2020 (*n* = 26; 31.7%), followed by 2022 (*n* = 16; 19.5%), 2023 (*n* = 14; 17.1%), 2021 (*n* = 12; 14.6%), and 2025 (*n* = 9; 11.0%), whereas 2024 accounted for three recoveries (3.7%) and only single WHO-priority recoveries were recorded in 2019 and 2026 (*n* = 1 each; 1.2%). When considered relative to all yeast-positive records submitted in each year, the proportion of WHO-priority recoveries was highest in 2019 (1/1; 100.0%) and 2025 (9/11; 81.8%), followed by 2023 (14/27; 51.9%), 2024 (3/7; 42.9%), 2022 (16/42; 38.1%), 2020 (26/77; 33.8%), 2026 (1/4; 25.0%), and 2021 (12/50; 24.0%), although estimates for years with very small denominators should be interpreted cautiously. The Fisher–Freeman–Halton exact test indicated a statistically significant association between year of submission and WHO-priority recovery (*p* = 0.005).

Although higher proportions of WHO-priority recoveries were observed in some of the more recent years, particularly 2023 and 2025, the year-to-year pattern was irregular and should be interpreted as temporal variation rather than a consistent increasing trend. Nevertheless, in an exploratory binary logistic regression model restricted to the years 2020–2025, year of submission remained significantly associated with WHO-priority recovery overall (likelihood ratio χ^2^ = 16.10, *df* = 5, *p* = 0.007), with significantly higher odds observed in 2023 (OR = 3.41, 95% CI: 1.26–9.23; *p* = 0.016) and 2025 (OR = 14.25, 95% CI: 2.70–75.22; *p* = 0.002), using 2021 as the reference category.

## 4. Discussion

A central finding of this study is that WHO critical- and high-priority yeasts account for a substantial proportion of veterinary yeast recoveries in Portugal. Rather than representing sporadic detections, these isolates indicate that medically important fungal taxa are detectable within animal-associated clinical diagnostic contexts, thereby highlighting a neglected veterinary component of the broader One Health landscape of fungal disease. In this context, veterinary diagnostic laboratories could, if linked to standardised identification and susceptibility-testing workflows, function as sentinel nodes within integrated European fungal surveillance systems, particularly for the early detection of ecological shifts and emerging resistance patterns.

### 4.1. Alignment with the WHO Fungal Priority Pathogens Framework

The WHO FPPL [11], published in 2022 and subsequently refined through systematic reviews and expert commentary, prioritises 19 fungal pathogens based on public health impact, mortality, incidence, antifungal resistance, diagnostic gaps and treatment availability. *C. albicans* and *C. neoformans* are classified as critical-priority pathogens, whereas *N. glabratus*, *C. parapsilosis* and *C. tropicalis* are listed as high-priority yeasts, reflecting their role in invasive candidiasis and cryptococcosis, their capacity to cause outbreaks, and their association with complex resistance phenotypes in human medicine. However, the FPPL was conceptualised primarily from a clinical and epidemiological viewpoint centred on human invasive disease, and relatively little structured attention has been devoted to the occurrence of these yeasts in animals and in non-clinical reservoirs, despite repeated calls from medical mycology and One Health authors to broaden surveillance beyond hospitals [6,10,34,35,36].

The absence of *C. auris* in the present dataset is noteworthy but should be interpreted cautiously. In fact, *C. auris* is a WHO critical-priority fungal pathogen because of its multidrug resistance, environmental persistence, ability to cause healthcare-associated outbreaks and infection-control challenges. However, its primary epidemiological niche remains strongly associated with human healthcare environments, particularly hospitals, intensive care units and long-term care settings. Recent European surveillance shows that *C. auris* is expanding rapidly across healthcare systems, with increasing case numbers and documented regional endemicity in several countries [37]. In Portugal, published reports remain comparatively limited, with the first documented Portuguese cases described in human healthcare settings in the year 2023 [38]. Therefore, the lack of *C. auris* detection in this veterinary diagnostic dataset most likely reflects the nature of the sampled animal populations, the passive diagnostic design and the still limited documented circulation of this pathogen in Portugal during the study period, rather than definitive absence from animal-associated or environmental reservoirs.

The predominance of *C. albicans* in our series broadly mirrors its global prominence as a leading cause of candidiasis in humans, while the frequent recovery of *N. glabratus* and *C. parapsilosis* parallels the increasing relevance of non-albicans *Candida* in European candidaemia cohorts. Nevertheless, the present data arise from diagnostic submissions rather than population-based surveillance and therefore provide insight into clinical suspicion and testing patterns as much as into the underlying epidemiology of colonisation and disease [34,39,40].

### 4.2. Ornamental Birds as Sentinels and Hosts

A salient feature of this study is the marked predominance of avian submissions, especially ornamental and companion birds, among WHO-priority yeast recoveries [41,42,43,44,45,46,47,48,49]. This predominance may, however, also be partly influenced by sampling bias, since ornamental and companion birds are more frequently represented in veterinary diagnostic submissions than other avian populations. This pattern is consistent with long-standing evidence that domestic and ornamental birds can act both as hosts and environmental sources for *C. neoformans* and related complexes, with faecal contamination of cages and surrounding environments contributing to the maintenance and dispersal of infectious propagules in proximity to humans [8,42]. Historical work in Europe has demonstrated high contamination rates with *C. neoformans* var. *neoformans* in breeding stocks of companion birds, with serovar distributions overlapping those observed in human cryptococcosis, thereby supporting the concept that ornamental birds may form part of a shared exposure network for cryptococcal infection [50,51].

Beyond cryptococci, ornamental birds are frequently colonised by a diversity of yeasts, including *Candida* species, which may occur as commensals in the gastrointestinal and upper respiratory tracts or manifest as clinical disease in the context of immunosuppression, stress, malnutrition, or concurrent infections [42]. In the present series, FPPL-listed yeasts were recovered from both clinically affected birds and from samples in which the distinction between colonisation and disease was not straightforward, underscoring the diagnostic challenge of interpreting yeast isolation in avian medicine. From a Planetary Health and One Health perspective, ornamental birds occupy highly anthropised niches: they share indoor environments with humans, are exposed to domestic micro-ecosystems shaped by cleaning agents and antimicrobial use and can interface with outdoor wildlife and urban bioaerosols. As such, ornamental birds may represent useful sentinel populations for shared environmental exposure to medically important yeasts [50,52].

### 4.3. Companion Animals, Marine Mammals and the Broader Veterinary Context

In addition to birds, WHO-priority yeasts in this study were recovered from dogs, cats and marine mammals, reflecting the broad host range of these organisms and echoing previous European and global reports of yeast colonisation and infection in companion animals and wildlife. Recent work in veterinary dentistry and oral microbiology has documented *C. albicans* and non-*albicans Candida* in the oral cavities of dogs and cats, often as low-prevalence colonisers, but with evidence of biofilm formation, virulence determinants and variable antifungal susceptibility, raising concerns about their potential to act as reservoirs for resistant strains [53]. Particularly for *Candida* spp., the emergence of azole resistance in animal-associated isolates may have important implications for human clinical management, given the likelihood of shared environmental reservoirs. Like birds, these domestic animals share the environment with humans, facilitating the transmission and dispersion of these pathogens [54,55,56].

Marine mammals represent another interface between environmental and clinical mycology, as they are exposed to aquatic and coastal ecosystems influenced by anthropogenic contamination, climate change and complex microbiota, yet systematic data on FPPL-listed yeasts in these hosts remain scarce. The isolation of critical- and high-priority yeasts from marine mammal submissions in the present study, although in small numbers, reinforces the notion that fungal threats span terrestrial and marine systems and that veterinary diagnostics can detect such occurrences before they are visible in human clinical data [57,58,59,60]. Given the very small number of marine mammal records, these findings should be interpreted as notable detections rather than evidence of a stable host-associated pattern.

### 4.4. One Health, Planetary Health and Antifungal Resistance

Emerging fungal diseases and antifungal resistance have been repeatedly framed as paradigmatic One Health and Planetary Health challenges, as they are driven by interacting forces such as climate change, global trade, agricultural practices, antimicrobial use, and alterations in wildlife and ecosystem health. European and international agencies have specifically warned that the use of azole fungicides in agriculture and horticulture, together with azole use in veterinary and human medicine, can select for azole-resistant *Aspergillus* and other fungi in the environment, with subsequent implications for human treatment failure [61,62]. Although the present study did not include systematic antifungal susceptibility testing, the detection of FPPL-listed yeasts in animals living in shared human environments supports the rationale for future susceptibility testing of animal-derived FPPL-listed yeasts, particularly in hosts sharing domestic or clinical environments with humans [14,35,61,63,64].

From a therapeutic perspective, the WHO-priority yeasts detected in this study are clinically relevant because they are associated with antifungal classes that are also central to human medicine, including azoles, echinocandins, polyenes and pyrimidine analogues. In human clinical practice, azoles such as fluconazole and voriconazole, echinocandins such as caspofungin, micafungin and anidulafungin, amphotericin B formulations and flucytosine remain key therapeutic options, depending on the fungal species, anatomical site and disease severity [11]. However, resistance patterns differ substantially among taxa: *N. glabratus* is particularly relevant because of reduced azole susceptibility and emerging echinocandin resistance; *C. tropicalis* has shown increasing azole resistance in several regions; the *C. parapsilosis* complex is characterised by comparatively reduced echinocandin susceptibility; and *C. auris*, although not detected here, remains one of the most important multidrug-resistant yeasts globally. Therefore, future veterinary surveillance of FPPL-listed yeasts should include antifungal susceptibility testing whenever clinically and epidemiologically appropriate [14,37,38,61,62].

Planetary Health emphasises that human health ultimately depends on the integrity of natural systems and that disturbances in climate, land use, biodiversity and pollution can reshape infectious disease patterns across species [65,66,67,68,69,70,71]. In this light, the occurrence of critical- and high-priority yeasts in ornamental birds, companion animals and marine mammals in Portugal should not be viewed solely as a veterinary diagnostic curiosity, but as part of a broader pattern in which fungal pathogens traverse human, animal and environmental domains [1,72]. Integrating veterinary mycology into fungal surveillance frameworks can help identify emerging reservoirs, track shifts in species prevalence and resistance, and support more sustainable antifungal stewardship across all sectors. This aligns with recent calls to operationalise One Health for fungal diseases by linking human clinical mycology with veterinary, wildlife and environmental monitoring.

### 4.5. Temporal Patterns and Interpretative Challenges

The temporal distribution of WHO-priority recoveries in this study was characterised by an irregular year-to-year pattern, with a numerical peak in 2020 and higher proportions of FPPL-listed yeasts in 2019 and 2025 relative to all yeast-positive submissions. Although exploratory regression suggested increased odds of WHO-priority recovery in 2023 and 2025 compared with 2021, the absence of a consistent trend and the small denominators in several years indicate that these fluctuations likely reflect variation in submission patterns, case mix and clinical suspicion rather than a clear secular increase in FPPL-listed yeasts. Moreover, the COVID-19 pandemic period overlaps partially with the study timeframe and may have influenced veterinary consultation and sampling behaviours in ways that are difficult to disentangle retrospectively, as has been reported for human fungal infections in Europe [10,35].

Interpreting yeast isolation from veterinary samples also poses intrinsic challenges. Many of the species identified, particularly *C. albicans* and *N. glabratus*, can behave as commensals or colonisers in mucosal and cutaneous sites, and their detection does not automatically equate to clinically relevant infection or zoonotic risk. The present data therefore document occurrence rather than incidence, and they cannot distinguish with certainty between colonisation, opportunistic infection, and incidental findings, especially when comprehensive clinical follow-up information is lacking. Nevertheless, even when colonising, the presence of FPPL-listed yeasts in animals contributes to the broader picture of host range, ecological niches and potential environmental dissemination pathways, which are all relevant for One Health risk assessment.

The non-WHO taxa identified in this study should not be regarded as clinically irrelevant solely because they are not included in the WHO FPPL. Rather, they represent a heterogeneous group of yeasts with variable ecological niches and pathogenic potential. Some may act predominantly as commensals, environmental organisms or incidental colonisers, whereas others may behave as opportunistic pathogens under specific host conditions, particularly in animals with altered mucosal barriers, dysbiosis, antimicrobial exposure, immunosuppression or concurrent disease [41,44,45,46,48,73]. In this context, taxa such as *P. laurentii*, *D. hansenii*, the *S. ciferrii* complex, *M. guilliermondii*, *C. sake* and *W. anomalus* should be interpreted as part of the broader microbiological background of veterinary diagnostic submissions. Their inclusion in the Supplementary Materials provides transparency and ecological context, but detailed species-specific pathogenicity assessment was beyond the predefined scope of this WHO-priority-focused study.

### 4.6. Methodological Limitations

Several methodological limitations should be acknowledged when interpreting these findings. First, this study was retrospective and laboratory-based, relying on clinical submissions rather than systematic sampling. Consequently, the data are subject to selection bias and cannot be used to estimate population-level prevalence or incidence of FPPL-listed yeasts in Portuguese animals. Moreover, as all included animals were sampled within a clinical diagnostic context, the findings should not be interpreted as evidence of subclinical carriage in the general animal population.

Second, the spectrum of host species and sample types reflects local referral patterns and the expertise of the participating veterinary laboratories, which may limit representativeness at the national level and restrict generalisability to other regions or practice settings in Portugal and Europe. In addition, yeast recovery does not necessarily imply clinically relevant fungal disease or direct zoonotic risk, as several of these organisms may act as commensals, colonisers or opportunistic pathogens depending on the host, anatomical site, immune status and clinical context.

Third, although yeast identification was performed using validated automated and MALDI-TOF MS methods, misidentification remains possible for closely related taxa, particularly within complexes such as *C. parapsilosis* sensu lato, and molecular confirmation was not systematically undertaken.

Fourth, this study did not include antifungal susceptibility testing or genotyping, precluding the assessment of resistance profiles and clonal relationships between animal isolates and human clinical strains reported in European FPPL-focused studies. The absence of antifungal susceptibility testing also reflects current veterinary diagnostic practice, in which antifungal susceptibility tests are rarely requested for yeast isolates, but highlights an important area for future routine implementation.

Finally, incomplete clinical information for some submissions, including details on immune status, comorbidities, previous antimicrobial or antifungal exposure, husbandry conditions, treatment protocols and clinical outcomes, limited the ability to correlate laboratory findings with disease severity, therapeutic response, recurrence or prognosis. This limitation is particularly relevant for avian submissions, in which prior antimicrobial exposure, medication administered through drinking water or feed, stress and husbandry conditions may influence intestinal dysbiosis and opportunistic yeast overgrowth.

### 4.7. Future Directions

Despite these limitations, the present study underscores the potential value of veterinary mycology in informing fungal surveillance and One Health strategies in Portugal and Europe. Future research should prioritise prospective, population-based studies of FPPL-listed yeasts in key animal hosts, including ornamental birds, companion animals and wildlife, with systematic collection of clinical data to distinguish colonisation from disease and to quantify the burden of clinically significant infections. Integrating veterinary laboratories into national and European fungal surveillance networks would facilitate the sharing of species distribution, antifungal susceptibility and resistance data across human, animal, and environmental sectors, in line with recent One Health recommendations from European agencies.

At the same time, targeted studies on antifungal susceptibility and resistance mechanisms in yeasts isolated from animals and their environments are needed to clarify potential links with human clinical strains and to evaluate the impact of cross-sectoral azole and other antifungal exposures. Given the strong representation of ornamental birds in the present dataset, multidisciplinary projects that combine veterinary clinical monitoring, environmental sampling of aviaries and households, and human exposure assessment would be particularly informative for operationalising One Health and Planetary Health surveillance in veterinary mycology [34,35,50].

A practical way to operationalise this surveillance would be to adapt the DAMA framework (Document, Assess, Monitor and Act) to veterinary mycology. In this context, veterinary laboratories would first document the occurrence of FPPL-listed and emerging non-WHO yeasts, assess their clinical and ecological relevance, monitor temporal, host-associated and resistance trends, and act through targeted communication with clinicians, public health authorities and environmental surveillance networks [74]. This approach would transform passive laboratory records into structured sentinel data and would align veterinary mycology with anticipatory One Health and Planetary Health surveillance [62].

## 5. Conclusions

This study illustrates that WHO critical- and high-priority yeasts were regularly detected among yeast-positive veterinary diagnostic submissions in Portugal between 2019 and 2026. The detected taxa included *C. albicans*, *N. glabratus*, *C. neoformans* var. *neoformans*, *C. parapsilosis* complex and *C. tropicalis*, recovered from avian hosts, dogs, cats and marine mammals. The predominance of ornamental birds in which these yeasts occur both as commensals and as agents of clinical disease supports the hypothesis that these species may serve as important sentinels at the interface between household microenvironments, urban ecosystems and wildlife. Collectively, these findings show that core taxa included in the WHO FPPL are already embedded in everyday veterinary practice, even though current prioritisation remains largely human-centred.

Within a One Health and Planetary Health framework, these findings argue that FPPL-listed yeasts should be regarded as shared pathogens of coupled human–animal–environment systems, whose emergence, adaptation and resistance are co-shaped by medical, veterinary, agricultural and environmental drivers. Recognising the contribution of veterinary laboratories to fungal surveillance, and systematically integrating animal mycological data, including from ornamental birds, into national and international monitoring networks would strengthen early detection of ecological shifts and resistance trends, and reposition veterinary mycology as an essential pillar of preparedness for fungal threats beyond human medicine.

## Figures and Tables

**Table 1:** Distribution of WHO critical- and high-priority fungal pathogens across animal host groups in Portugal, 2019–2026.

Distribution of WHO Critical- and High-Priority Fungal Pathogens by Animal Host Groups							
	WHO-Priority			Non-WHO			Total
Host Group	*n*	% Within Host Group	95% CI (%)	*n*	% Within Host Group	95% CI (%)	*n*
Avian	57	37.5%	29.8–45.7	95	62.5%	54.3–70.2	152
Canine	12	34.3%	19.1–52.2	23	65.7%	47.8–80.9	35
Feline	8	40%	19.1–63.9	12	60.0%	36.1–80.9	20
Equine	0	0.0%	0.0–84.2	2	100%	15.8–100	2
Lagomorphs	0	0.0%	0.0–97.5	1	100%	2.5–100	1
Marine mammals	5	100%	47.8–100	0	0.0%	0.0–52.2	5
Non-mammalian non-avian vertebrates	0	0.0%	0.0–60.2	4	100%	39.8–100.0	4
**Total**	82	37.4%	31.0–44.2	137	62.6%	55.8–69.0	219

CI, confidence interval; Non-mammalian non-avian vertebrates include reptiles and fish; WHO, World Health Organization.

**Table 2:** Distribution of WHO-priority and non-WHO fungal pathogens by sample type, Portugal, 2019–2026.

Distribution of WHO Critical- and High-Priority Fungal Pathogens by Samples					
	WHO-Priority		Non-WHO		Total
Sample Type	*n*	% Within Sample Type	*n*	% Within Sample Type	*n*
Faecal swab	43	35.3%	79	64.8%	122
Cutaneous swab	15	57.7%	11	42.3%	26
Oropharyngeal swab	12	70.6%	5	29.4%	17
Ear swab	4	28.6%	10	71.4%	14
Organ tissue sample	3	27.3%	8	72.7%	11
Nasal/ocular swab	1	9.1%	10	90.9%	11
Urine	2	28.6%	5	71.4%	7
Egg content sample	0	0.0%	5	100%	5
Uterine swab	0	0.0%	3	100%	3
Air sac swab	1	50.0%	1	50%	2
Spiracle swab	1	100%	0	0.0%	1
**Total**	82	37.4%	137	62.6%	219

**Table 3:** Distribution of WHO critical- and high-priority fungal pathogens by mammalian species, 2019–2026.

		Critical Priority		High Priority			
Host Species	Species	*Cryptococcus* *neoformans* var. *neoformans* *n* (%)	*Candida* *albicans* *n* (%)	*Nakaseomyces glabratus* *n* (%)	*Candida* *parapsilosis* Complex *n* (%)	*Candida tropicalis* *n* (%)	Total
Feline	European domestic cat	4 (57.1%)	0 (0.0%)	0 (0.0%)	3 (42.9%)	0 (0.0%)	7
	Persian	0 (0.0%)	1 (100%)	0 (0.0%)	0 (0.0%)	0 (0.0%)	1
Canine	Mixed-breed	5 (62.5%)	1 (12.5%)	0 (0.0%)	1 (12.5%)	1 (12.5%)	8
	Boston Terrier	2 (100%)	0 (0.0%)	0 (0.0%)	0 (0.0%)	0 (0.0%)	2
	German Shepherd	1 (50.0%)	0 (0.0%)	0 (0.0%)	1 (50.0%)	0 (0.0%)	2
Marine mammals	Common dolphin (*Delphinus delphis*)	0 (0.0%)	3 (60.0%)	2 (40.0%)	0 (0.0%)	0 (0.0%)	5
**Total**		12 (48.0%)	5 (20.0%)	2 (8.0%)	5 (20.0%)	1 (4.0%)	25

**Table 4:** Distribution of WHO critical- and high-priority fungal pathogens by avian species, 2019–2026.

	Critical Priority	High Priority			
Avian Species	*Candida* *albicans* *n* (%)	*Nakaseomyces glabratus* *n* (%)	*Candida parapsilosis* Complex *n* (%)	*Candida* *tropicalis* *n* (%)	Total
African grey parrot *(Psittacus erithacus)*	5 (71.4%)	1 (14.3%)	1 (14.3%)	0 (0.0%)	7
Alexandrine parakeet *(Psittacula eupatria)*	0 (0.0%)	3 (100%)	0 (0.0%)	0 (0.0%)	3
Blue-and-yellow macaw *(Ara ararauna)*	0 (0.0%)	2 (100%)	0 (0.0%)	0 (0.0%)	2
Bluebonnet *(Northiella haematogaster)*	1 (100%)	0 (0.0%)	0 (0.0%)	0 (0.0%)	1
Blue-fronted amazon *(Amazona aestiva xanthopteryx)*	0 (0.0%)	1 (100%)	0 (0.0%)	0 (0.0%)	1
Blue-winged parrot *(Neophema chrysostoma)*	1 (100%)	0 (0.0%)	0 (0.0%)	0 (0.0%)	1
Budgerigar *(Melopsittacus undulatus)*	0 (0.0%)	1 (100%)	0 (0.0%)	0 (0.0%)	1
Channel-billed toucan *(Ramphastos vitellinus)*	1 (100%)	0 (0.0%)	0 (0.0%)	0 (0.0%)	1
Cockatiel *(Nymphicus hollandicus)*	5 (50.0%)	4 (40.0%)	1 (10.0%)	0 (0.0%)	10
Common pheasant *(Phasianus colchicus)*	1 (100%)	0 (0.0%)	0 (0.0%)	0 (0.0%)	1
Domestic canary *(Serinus canaria)*	3 (75.0%)	1 (25.0%)	0 (0.0%)	0 (0.0%)	4
Eastern rosella *(Platycercus eximius)*	1 (100%)	0 (0.0%)	0 (0.0%)	0 (0.0%)	1
Galah *(Eolophus roseicapilla)*	1 (100%)	0 (0.0%)	0 (0.0%)	0 (0.0%)	1
Gouldian finch *(Chloebia gouldiae)*	1 (50.0%)	1 (50.0%)	0 (0.0%)	0 (0.0%)	2
Green-winged macaw *(Ara chloropterus)*	1 (50.0%)	1 (50.0%)	0 (0.0%)	0 (0.0%)	2
Lovebird *(Agapornis roseicollis)*	5 (71.4%)	1 (14.3%)	1 (14.3%)	0 (0.0%)	7
Major Mitchell’s cockatoo *(Lophochroa leadbeateri)*	1 (100%)	0 (0.0%)	0 (0.0%)	0 (0.0%)	1
Meyer’s parrot *(Poicephalus meyeri)*	0 (0.0%)	1 (100%)	0 (0.0%)	0 (0.0%)	1
Rainbow lorikeet *(Trichoglossus moluccanus)*	1 (100%)	0 (0.0%)	0 (0.0%)	0 (0.0%)	1
Red-browed amazon *(Amazona rhodocorytha)*	0 (0.0%)	0 (0.0%)	0 (0.0%)	1 (100%)	1
Red-tailed black cockatoo *(Calyptorhynchus banksii)*	1 (100%)	0 (0.0%)	0 (0.0%)	0 (0.0%)	1
Rose-ringed parakeet *(Psittacula krameri)*	1 (100%)	0 (0.0%)	0 (0.0%)	0 (0.0%)	1
Western bluebill *(Spermophaga haematina)*	1 (100%)	0 (0.0%)	0 (0.0%)	0 (0.0%)	1
White cockatoo *(Cacatua alba)*	2 (66.7%)	1 (33.3%)	0 (0.0%)	0 (0.0%)	3
Yellow-crowned amazon *(Amazona ochrocephala)*	0 (0.0%)	0 (0.0%)	1 (100%)	0 (0.0%)	1
Yellow-crowned parakeet *(Cyanoramphus auriceps)*	0 (0.0%)	0 (0.0%)	1 (100%)	0 (0.0%)	1
**Total**	33 (57.9%)	18 (31.6%)	5 (8.8%)	1 (1.8%)	57

Percentages are calculated within each avian host species. Totals represent the proportion of each fungal pathogen and taxonomically updated related species among all positive avian cases (*n* = 57).

## Data Availability

The data presented in this study are available from the corresponding author upon reasonable request. Restrictions apply because the dataset contains anonymised veterinary diagnostic records derived from clinical submissions.

## Supplementary Materials

The following supporting information can be downloaded at: https://www.mdpi.com/article/10.3390/pathogens15070660/s1, Table S1. Distribution of non-WHO-prioritised yeast taxa by broad animal class in veterinary diagnostic submissions from Portugal, 2019–2026.

## Abbreviations

The following abbreviations are used in this manuscript:

FPPL	Fungal Priority Pathogens List
MALDI-TOF MS	Matrix-assisted laser desorption/ionisation time-of-flight mass spectrometry
WHO	World Health Organization
