# Supplementary material for: WHO Critical- and High-Priority Fungal Pathogens Beyond Human Medicine: Expanding One Health Surveillance
Source: Pathogens. 2026 Jun 23;15(7):660. doi: 10.3390/pathogens15070660 (PMC13415207; doi:10.3390/pathogens15070660)
Supplement: Supplementary file 1 [file pathogens-15-00660-s001.zip › pathogens-4379364-supplementary.pdf]

Table S1. Distribution of non-WHO-prioritised yeast taxa by broad animal class in veterinary diagnostic submissions from Portugal, 2019–2026.

Taxon	Animal Class							
	Birds		Mammals		Others		Total	
	n	%	*n*	%	*n*	%	*n*	%
Papiliotrema laurentii (formerly *Cryptococcus laurentii*)	17	12.4%	4	2.9%	0	0.0%	21	15.3%
Debaryomyces hansenii (formerly *Candida famata*)	16	11.7%	5	3.7%	0	0.0%	21	15.3%
Stephanoascus ciferrii complex (formerly *Candida ciferrii*)	7	5.1%	7	5.1%	1	0.7%	15	11.0%
Meyerozyma guilliermondii (formerly *Candida guilliermondii*)	7	5.1%	5	3.7%	0	0.0%	12	8.8%
Candida sake	9	6.6%	2	1.5%	1	0.7%	12	8.8%
Wickerhamomyces anomalus (formerly *Candida pelliculosa*)	4	2.9%	5	3.7%	0	0.0%	9	6.6%
Clavispora lusitaniae (formerly *Candida lusitaniae*)	6	4.4%	0	0.0%	0	0.0%	6	4.4%
Rhodotorula glutinis	2	1.5%	2	1.5%	1	0.7%	5	3.7%
Candida zeylanoides	5	3.7%	0	0.0%	0	0.0%	5	3.7%
Rhodotorula mucilaginosa	2	1.5%	2	1.5%	0	0.0%	4	2.9%
Diutina catenulata (formerly *Candida catenulata*)	4	2.9%	0	0.0%	0	0.0%	4	2.9%
Malassezia furfur	0	0.0%	3	2.2%	0	0.0%	3	2.2%
Sungouiella intermedia (formerly *Candida intermedia*)	1	0.7%	0	0.0%	1	0.7%	2	1.5%
Starmerella magnoliae (formerly *Candida magnoliae*)	2	1.5%	0	0.0%	0	0.0%	2	1.5%
Saccharomyces cerevisiae	2	1.5%	0	0.0%	0	0.0%	2	1.5%
Kluyveromyces lactis (formerly *Candida sphaerica*)	1	0.7%	1	0.7%	0	0.0%	2	1.5%
Cyberlindnera jadinii (formerly *Candida utilis*)	1	0.7%	0	0.0%	0	0.0%	1	0.7%
Trichosporon asahii	1	0.7%	0	0.0%	0	0.0%	1	0.7%
Torulaspora delbrueckii (formerly *Candida colliculosa*)	1	0.7%	0	0.0%	0	0.0%	1	0.7%
Diutina rugosa (formerly *Candida rugosa*)	1	0.7%	0	0.0%	0	0.0%	1	0.7%
Pichia norvegensis (formerly *Candida norvegensis*)	1	0.7%	0	0.0%	0	0.0%	1	0.7%
Hanseniaspora spp (formerly *Kloeckera* spp.)	1	0.7%	0	0.0%	0	0.0%	1	0.7%
Solicoccozyma terrea (formerly *Cryptococcus terreus*)	1	0.7%	0	0.0%	0	0.0%	1	0.7%
Malassezia pachydermatis	0	0.0%	1	0.7%	0	0.0%	1	0.7%
Candida dubliniensis	1	0.7%	0	0.0%	0	0.0%	1	0.7%
Geotrichum klebahnii	0	0.0%	1	0.7%	0	0.0%	1	0.7%
Cystobasidium minutum (formerly *Rhodotorula minuta*)	1	0.7%	0	0.0%	0	0.0%	1	0.7%
Metschnikowia pulcherrima (formerly *Candida pulcherrima*)	1	0.7%	0	0.0%	0	0.0%	1	0.7%
**Total**	95	69.3%	38	27.7%	4	2.9%	137	100%

Percentages are calculated relative to the total number of non-WHO-prioritised yeast isolates (*n* = 137). The "Others" category includes non-mammalian, non-avian vertebrates. WHO, World Health Organization.
